# Secondary hyperparathyroidism and lower hip bone mineral density in very old hospitalized adults: a real-world endocrine bone study

**DOI:** 10.1186/s12902-026-02456-y

**Published:** 2026-08-06

**Authors:** Stylianos Kopanos, Sandra Scheel, Bettina Eggert, Alexander Rübberdt, Christiane Muth, Ulrich Thiem, Joachim Feldkamp

**Affiliations:** 1 Academic Department of General Internal Medicine, Endocrinology, Diabetes and Infectiology, Bielefeld University, Medical School and University Medical Center East Westphalia-Lippe, Bielefeld, Germany; 2 Department of Orthopaedics and Trauma Surgery, University Medical Center East Westphalia-Lippe, University of Bielefeld, Bielefeld, Germany; 3 Department of General Practice and Family Medicine, Medical School OWL, University Bielefeld, Bielefeld, Germany; 4 Department of Geriatrics, Bielefeld University, Medical School and University Medical Center OWL, Klinikum Bielefeld, Bielefeld, Germany

**Keywords:** Osteoporosis, Bone Mineral Density (BMD), Thyroid Function, Parathormone, Vitamin D, Mobility

## Abstract

**Background:**

Population ageing is associated with increasing rates of osteoporosis, frailty, and functional decline. Vitamin D deficiency and disturbances in calcium–parathyroid hormone (PTH) homeostasis are common in older adults and may contribute to bone loss. However, real-world data describing the frequency and skeletal relevance of these endocrine abnormalities in very old hospitalized populations remain limited.

**Materials and methods:**

We conducted a retrospective cross-sectional study including 1,202 hospitalized patients aged ≥70 years (mean age 85.0 ± 7.1 years) who underwent dual-energy X-ray absorptiometry at a tertiary endocrine and geriatric centre in Germany. Serum 25-hydroxyvitamin D, calcium, parathyroid hormone (PTH), thyroid-stimulating hormone (TSH), and renal function parameters were extracted from electronic medical records. Hip bone mineral density (BMD) served as the primary skeletal outcome. Multivariable linear regression models were used to assess associations between endocrine parameters and BMD after adjustment for age, sex, body mass index, and living setting.

**Results:**

Vitamin D deficiency was highly prevalent in this very old cohort. Vitamin D levels <20 ng/mL were present in 68.6% of participants with available vitamin D measurements, and levels <10 ng/mL in 45.5%. Hypocalcaemia was observed in 39.1% of participants, while elevated PTH levels occurred in 29.9%. The combination of hypocalcaemia and elevated PTH—consistent with secondary hyperparathyroidism—was identified in 13.7% of patients. Participants with elevated PTH had significantly lower hip BMD compared with those without PTH elevation (median −2.3 vs −1.9, p = 0.004). This association remained significant after adjustment for age and sex (β −0.23, 95% CI: −0.45 to −0.01; p = 0.042). In multivariable models, increasing age (β −0.046 per year, 95% CI: −0.063 to −0.029; p < 0.001) and higher PTH levels (β −0.006 per unit, 95% CI: −0.010 to −0.002; p = 0.002) were independently associated with lower hip BMD. In contrast, vitamin D supplementation (β = −0.113, 95% CI −0.493 to 0.266; p = 0.56) and calcium supplementation (β = 0.101, 95% CI −0.556 to 0.758; p = 0.76) were not independently associated with BMD. Living setting was also not independently associated with hip BMD after adjustment (β −0.326, 95% CI: −0.858 to 0.207; p = 0.23). Exploratory analyses suggested a U-shaped relationship between TSH and hip BMD, with both suppressed and elevated TSH levels associated with lower BMD compared with the euthyroid range.

**Conclusion:**

Severe vitamin D deficiency is common in very old hospitalized patients and frequently coexists with hypocalcaemia and secondary hyperparathyroidism. Elevated PTH levels are independently associated with lower hip bone mineral density, highlighting the importance of evaluating the calcium–vitamin D–PTH axis. Both suppressed and elevated TSH levels were associated with lower BMD. These findings underscore the need for comprehensive endocrine assessment of bone health in geriatric patients.

**Supplementary Information:**

The online version contains supplementary material available at 10.1186/s12902-026-02456-y.

## Introduction

Population ageing represents one of the most pressing challenges for health care systems in high-income countries where the proportion of very old adults continues to increase [1]. With advancing age, the burden of musculoskeletal disorders rises substantially, leading to impaired mobility, loss of independence, increased fall risk, and a growing need for long-term care [2, 3]. Osteoporosis and reduced bone mineral density (BMD) play a central role in this context, as they are closely linked to fractures, functional decline, and excess morbidity in older adults [4].

Bone health in advanced age is strongly influenced by endocrine factors, most prominently vitamin D status, calcium homeostasis, and parathyroid hormone (PTH) regulation [5]. Vitamin D deficiency is highly prevalent among older adults, particularly in those with limited outdoor exposure, reduced mobility, or institutionalized living conditions [6]. Secondary hyperparathyroidism as a consequence of vitamin D deficiency may further accelerate bone loss and contribute to skeletal fragility [7]. Although the biological relationships between vitamin D, calcium, PTH, and bone metabolism are well established, real-world data from very old populations in contemporary care settings remain limited [8, 9].

In addition to skeletal outcomes, mobility and functional independence are key determinants of health-related quality of life and health care utilization in older adults. Measures such as the Timed Up and Go (TUG) test and the Barthel Index are widely used in clinical practice to assess mobility, functional performance, and the need for supportive care [10, 11]. Importantly, impaired mobility and reduced bone strength are closely interrelated, yet they are often studied separately. Integrating objective measures of mobility with bone health parameters may therefore provide clinically meaningful insights, particularly from a primary care and geriatric perspective [12].

Living setting represents a critical, but often underappreciated, determinant of bone health and functional status in older adults. Nursing home residents typically differ markedly from community-dwelling older adults with respect to physical activity, sun exposure, nutritional status, comorbidity burden, and medication use [13]. These differences may translate into distinct patterns of vitamin D deficiency, secondary hyperparathyroidism, reduced BMD, and impaired mobility. However, comparative data directly contrasting nursing home residents and independently living older adults within the same health care system are scarce, especially in very old cohorts [14, 15].

Thyroid function represents an additional endocrine factor that may influence bone health in older adults. Both overt and subclinical thyroid dysfunction, as well as thyroid hormone replacement or antithyroid therapy, have been associated with changes in bone turnover and fracture risk [16, 17]. In very old populations, interpretation of thyroid function parameters is further complicated by multimorbidity and medication use [18]. Nevertheless, evaluating thyroid function in conjunction with bone health parameters may add clinically relevant information when appropriately adjusted for treatment status [19].

Against this background, the present study aimed to assess the prevalence and skeletal relevance of disturbances in the vitamin D–calcium–parathyroid hormone (PTH) axis in a very old hospitalized population. In particular, we investigated the frequency of severe vitamin D deficiency, hypocalcaemia, and secondary hyperparathyroidism and their association with bone mineral density (BMD). Given the clinical heterogeneity of geriatric patients, we additionally compared endocrine and skeletal characteristics between nursing home residents and community-dwelling older adults. As a secondary exploratory objective, we evaluated thyroid function parameters and thyroid medication use in relation to BMD. By integrating endocrine parameters, bone mineral density measurements, and functional indicators in a large real-world geriatric cohort, this study aims to provide clinically relevant insights into endocrine determinants of skeletal health in advanced age.

## Materials and methods

### Study design & population

This study is a retrospective cross-sectional analysis conducted at the Academic Department of General Internal Medicine, Endocrinology, Diabetes, and Infectiology, Klinikum Bielefeld, Germany, a tertiary care centre specialized in endocrine and geriatric patient management. The study population consisted of 1,202 hospitalized patients aged 70 years or older who underwent bone mineral density (BMD) assessment over a period of seven years. Patients were identified and selected from electronic medical records, ensuring complete laboratory and densitometry data.

All available patients with documented information on living setting (community-dwelling vs. nursing home residence) and at least one relevant clinical outcome (bone mineral density and/or functional assessment) were eligible for inclusion. The primary aim was to characterize endocrine and functional correlates of bone health in advanced age and to compare skeletal and clinical parameters between community-dwelling older adults and nursing home residents.

The cohort consisted predominantly of orthogeriatric patients admitted with acute fracture-related diagnoses. However, fracture diagnoses were recorded as free-text clinical entries and were not systematically classified according to anatomical site, trauma mechanism, fragility status, or previous fracture history.

Demographic variables (age, sex) and routinely measured laboratory parameters were extracted from the medical records, including serum calcium, 25-hydroxyvitamin D, parathyroid hormone (PTH), and thyroid-related markers (TSH, free thyroxine [fT4], and free triiodothyronine [fT3] when available). Medication exposure variables were coded based on documentation and included vitamin D supplementation, calcium supplementation, and thyroid-related medication use. Osteoporosis-specific medications were not included because these data were not systematically recorded in the source database.

Bone mineral density (BMD) was assessed using dual-energy X-ray absorptiometry (DXA) where available, with hip BMD used as the primary skeletal outcome due to higher completeness and clinical relevance in this cohort. Lumbar spine BMD was considered secondary and described descriptively because of substantial missingness. Functional status was assessed using the Timed Up and Go (TUG) test (mobility) and the Barthel Index (activities of daily living) when available [20].

The study was conducted in accordance with the Declaration of Helsinki and was approved by the Aerztekammer Westfalen-Lippe Ethics committee (approval number 2025-443-f-S). Written informed consent to participate was obtained from all participants.

### Inclusion and exclusion criteria

Patients were eligible for inclusion if they had available clinical data during the study period and at least one relevant outcome of interest, including hip bone mineral density (BMD) assessment by dual-energy X-ray absorptiometry (DXA) and/or functional assessment. Routine laboratory data related to bone and endocrine metabolism, including serum calcium, parathyroid hormone (PTH), vitamin D, and thyroid-stimulating hormone (TSH), were included when available. Free thyroxine (fT4) and free triiodothyronine (fT3) were analysed in exploratory sub-analyses due to substantial missingness.

Patients were excluded if medical records were insufficient to determine key demographic variables or living setting. Individuals with conditions known to profoundly affect bone metabolism independent of age-related osteoporosis were excluded, including advanced chronic kidney disease (estimated glomerular filtration rate < 30 mL/min/1.73 m²), malignancies with bone metastases, and severe inflammatory or endocrine disorders affecting bone health (e.g., untreated overt hyperthyroidism or primary hyperparathyroidism). Patients receiving long-term systemic glucocorticoid therapy (> 5 mg prednisolone equivalent for more than 6 months) were also excluded.

Given the retrospective, real-world nature of the cohort, patients receiving vitamin D, calcium, or thyroid-related medications were not excluded, and medication use was explicitly accounted for in the analyses.

### Bone mineral density assessment

Hip bone mineral density (BMD) was assessed using dual-energy X-ray absorptiometry (DXA) with a Hologic Discovery scanner (Hologic Inc., Marlborough, MA, USA). All examinations were performed according to standardized institutional protocols by trained personnel following the manufacturer’s recommendations. Routine quality assurance procedures were performed as part of standard clinical practice. Hip BMD was selected as the primary skeletal outcome because it is clinically highly relevant in older adults and is less prone to artefactual distortion from age-related degenerative changes than lumbar spine measurements. In very old patients, lumbar spine BMD may be affected by osteophytes, vertebral deformities, scoliosis, and vascular calcifications, which can reduce measurement reliability and may lead to an overestimation of true bone density. Therefore, inferential analyses focused on hip BMD as the main densitometric parameter. BMD results were expressed as T-scores according to the World Health Organization criteria, with osteoporosis defined as a T-score ≤ − 2.5, osteopenia as a T-score between − 1.0 and − 2.5, and normal bone density as a T-score ≥ − 1.0 [21].

DXA measurements were performed as part of routine clinical care using standardized protocols and regular device calibration. Site-specific precision data and coefficients of variation for DXA and laboratory measurements were not available because of the retrospective nature of the study.

### Biochemical and laboratory measurements

Routine laboratory parameters were obtained from clinical records. Serum calcium, parathyroid hormone (PTH), and 25-hydroxyvitamin D were included as key markers of calcium–vitamin D metabolism. Secondary hyperparathyroidism was operationally defined as the combination of hypocalcaemia and elevated PTH (> 65 pg/mL). Thyroid-related parameters included thyroid-stimulating hormone (TSH) and, when available, free thyroxine (fT4) and free triiodothyronine (fT3). According to the institutional laboratory reference range, normal serum TSH concentrations were 0.27–4.20 mIU/L. Vitamin D supplementation, calcium supplementation, and thyroid-related medication use were coded based on documented treatment. Serum 25-hydroxyvitamin D and intact parathyroid hormone were measured in the central clinical laboratory using automated electrochemiluminescence immunoassays on a Cobas/Elecsys platform (Roche Diagnostics, Mannheim, Germany), according to the manufacturer’s instructions.

Given the retrospective, real-world nature of the data, laboratory measurements were interpreted in conjunction with medication use and clinical context rather than as isolated biomarkers.

### Study outcomes

The primary skeletal outcome was hip bone mineral density (BMD). Lumbar spine BMD (L1–L4) was considered a secondary descriptive outcome because of substantial missingness. Mobility was primarily assessed using the Timed Up and Go (TUG) test; functional independence was assessed using the Barthel Index as a secondary functional outcome.

The main endocrine exposures of interest were serum 25-hydroxyvitamin D [25(OH)D], calcium, and parathyroid hormone (PTH). Thyroid function parameters (TSH, and free T4 when available) were evaluated in secondary/exploratory analyses in relation to BMD.

### Statistical analysis

#### Data description and group comparisons

Analyses were primarily conducted as complete-case analyses because missingness primarily affected outcome variables (hip BMD and functional assessments), for which multiple imputation would have required strong assumptions regarding the missing data mechanism.

Continuous variables were assessed for distributional characteristics using visual inspection (histograms/Q–Q plots) and summary statistics. Normally distributed variables are presented as mean ± standard deviation (SD), and non-normally distributed variables as median (interquartile range, IQR). Categorical variables are presented as counts and percentages. These descriptive analyses provided an overview of the demographic and clinical composition of the cohort, highlighting potential differences in BMD across subgroups.

Participants were classified according to living setting (nursing home residents vs. community-dwelling older adults). Between-group comparisons were performed using Student’s t-test for approximately normally distributed continuous variables, the Mann–Whitney U test for non-normally distributed variables, and χ² or Fisher’s exact test for categorical variables as appropriate.

Medication information was extracted from the baseline medication list and coded into binary indicators for vitamin D supplementation and calcium supplementation. Thyroid therapy was coded as a categorical variable with the following levels: (i) thyroid hormone replacement (e.g., levothyroxine ± liothyronine), (ii) antithyroid drugs, and (iii) other thyroid-relevant medication (e.g., iodine or amiodarone); additionally, a binary indicator of thyroid hormone replacement therapy use was derived for descriptive analyses and sensitivity analyses.

Associations with BMD and mobility outcomes were examined using multivariable linear regression. Separate models were fitted for hip BMD and lumbar spine BMD (L1–L4). Core covariates included age, sex, body mass index (BMI, if available), and living setting (nursing home vs. community-dwelling). The primary endocrine predictors (25(OH)D, calcium, PTH) were entered simultaneously to assess their independent associations. Vitamin D and calcium supplementation indicators were included as covariates in robustness analyses. Adjusted odds ratios (ORs) and 95% confidence intervals (CIs) were reported to quantify the impact of each biochemical factor on osteoporosis risk. Regression diagnostics, including variance inflation factors (VIFs) and residual plots, were utilized to ensure model validity and check for issues such as multicollinearity or heteroscedasticity.

To assess functional relevance, an additional multivariable model was fitted with TUG as the dependent variable, including age, sex, BMI (if available), living setting, 25(OH)D, calcium, PTH, and BMD (hip or lumbar spine, examined in separate specifications). If TUG showed marked right-skewness, log-transformation was applied and results were interpreted accordingly.

To evaluate whether thyroid function added explanatory value beyond the core endocrine model, nested models were used. A base model for BMD included age, sex, BMI (if available), living setting, 25(OH)D, calcium, PTH, and supplementation variables. TSH (and free T4 where available) were then added to the base model together with thyroid medication category. Model fit and incremental contribution were assessed by comparing regression coefficients and confidence intervals, and—where appropriate—changes in explained variance or information criteria.

### Effect modification and exploratory analyses

To explore the fundamental (inter)relationships between key biochemical markers (thyroid hormones, vitamin D, parathyroid hormone [PTH], and renal function markers) and BMD, Pearson or Spearman correlation coefficients were calculated. The choice of correlation method was determined by the distribution of the data with Pearson correlation used for normally distributed variables and Spearman correlation applied for non-normally distributed variables or ordinal data. The results were visualized using scatter plots and correlation heatmaps.

### Sensitivity analyses

Sensitivity analyses were performed to assess robustness of findings: (i) repeating key models after excluding participants receiving vitamin D and/or calcium supplementation (confounding by indication), and (ii) repeating thyroid-related models after excluding participants receiving thyroid medication. Where extreme outliers were present in endocrine variables (e.g., TSH), analyses were repeated with influence diagnostics and/or robust approaches to ensure stability of estimates. Analyses were primarily conducted as complete-case analyses; the number of participants included in each model was reported. All tests were two-sided and p-values < 0.05 were considered statistically significant. Analyses were performed using RStudio (version 2024.12.0 + 467, Boston, MA, USA) for data processing, visualization, and modelling.

## Results

### Study population

**Fig. 1:**
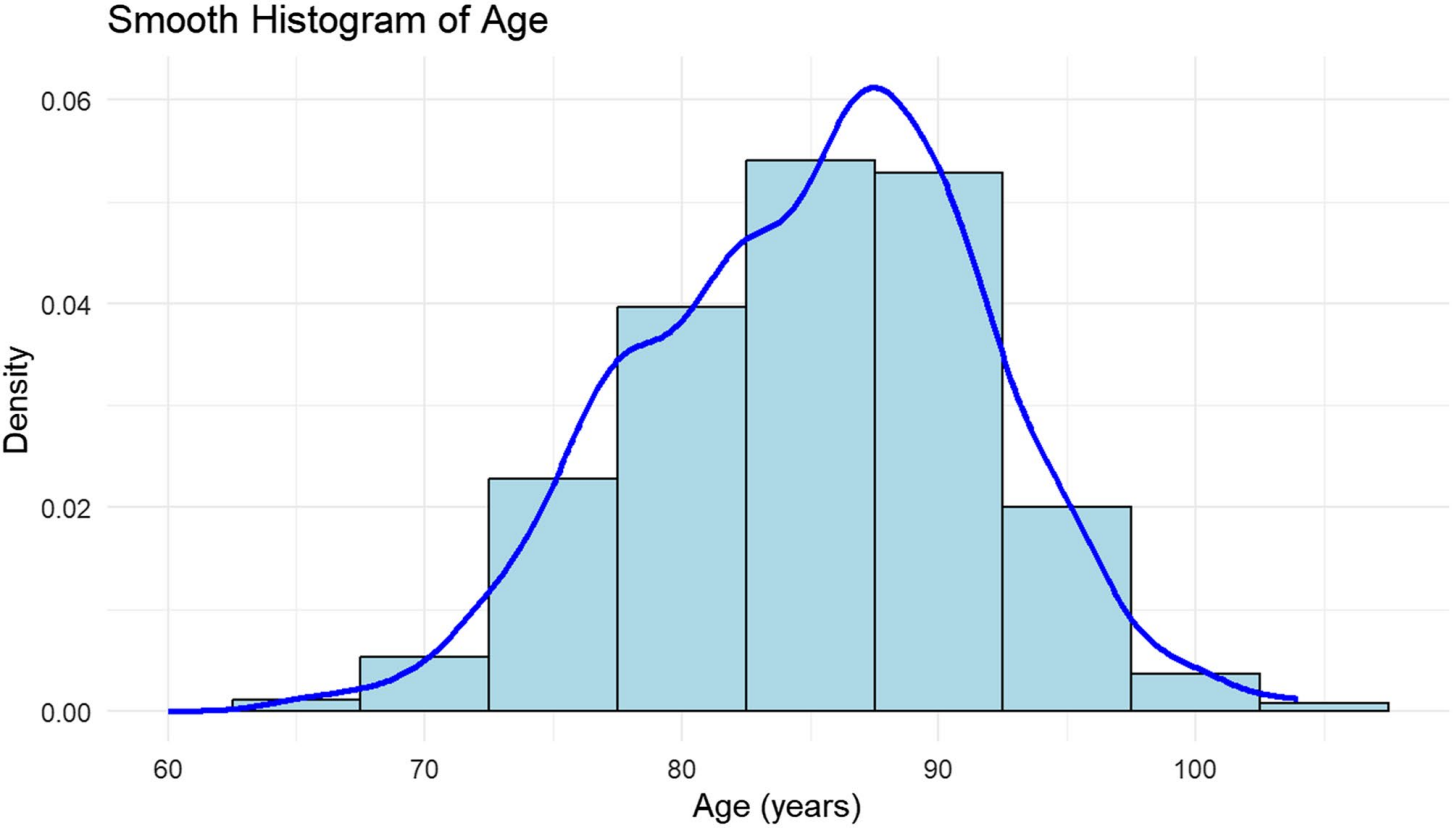
Smooth histogram of age distribution. This histogram provides a density-based representation of age distribution in the study population

A total of 1,202 participants were included in the analysis, comprising 1,029 community-dwelling older adults, 123 nursing home residents, and 50 participants with missing information on living setting. The study population was very old, with a mean age of 85.0 ± 7.1 years **(**Fig. 1**)**. Nursing home residents were significantly older than community-dwelling participants (mean 88 ± 6 vs. 85 ± 7 years, *p* < 0.001), while sex distribution was comparable between groups (*p* = 0.7).

## Discussion

In this large cohort of very old hospitalized patients, we observed a strikingly high prevalence of disturbances in the vitamin D–calcium–parathyroid hormone axis. More than two thirds of participants with available vitamin D measurements had vitamin D levels below 20 ng/mL and almost half had levels below 10 ng/mL, indicating severe vitamin D deficiency in a substantial proportion of this geriatric population. Importantly, this deficiency frequently coexisted with hypocalcaemia and elevated parathyroid hormone levels, resulting in secondary hyperparathyroidism in more than one in seven patients. Elevated PTH levels were independently associated with lower hip bone mineral density, suggesting that endocrine dysregulation of calcium homeostasis represents an important determinant of skeletal vulnerability in advanced age. These findings highlight that, in very old patients, assessment of bone health should extend beyond isolated vitamin D measurements and instead consider the broader calcium–vitamin D–PTH axis [22].

Contrary to common expectations, nursing home residence was not independently associated with lower hip BMD after adjustment for age, sex, and endocrine factors. This finding is clinically relevant, as nursing home residents are often presumed to be at uniformly higher risk of osteoporosis. Our results indicate that differences observed in unadjusted analyses may largely reflect age-related effects rather than living setting itself. This suggests that osteoporosis risk assessment and management should not rely solely on residential status. Community-dwelling older adults—who may appear more independent—can exhibit comparable skeletal vulnerability and therefore require equally systematic evaluation and preventive strategies. The focus on hip BMD represents a clinically appropriate approach in this very old inpatient cohort. Lumbar spine BMD can be difficult to interpret in advanced age because degenerative changes, osteophytes, vertebral deformities, and vascular calcifications may artefactually increase measured BMD and reduce its validity as a marker of skeletal fragility. Hip BMD was therefore considered the most robust densitometric parameter for the present analyses.

Importantly, supplementation status itself was not independently associated with hip BMD, supporting the interpretation that measured vitamin D levels may act as a marker of clinical attention rather than a direct causal determinant in this cohort. These findings highlight the need for cautious interpretation of vitamin D measurements in routine care of very old adults and argue against simplistic conclusions based on single laboratory values [5, 23]. Furthermore, detailed information regarding vitamin D supplementation, including dose, duration, formulation, and treatment adherence, was unavailable. Consequently, we were unable to assess dose-response relationships or determine whether supplementation met currently recommended doses for fracture prevention in older adults. These factors should be addressed in future prospective studies. In routine clinical practice, vitamin D supplementation is frequently prescribed to patients already considered at increased risk for osteoporosis or vitamin D deficiency. Therefore, confounding by indication may partly explain the absence of an independent association between supplementation and hip BMD.

In contrast to vitamin D, PTH showed a robust and biologically plausible association with hip BMD. Higher PTH levels were independently associated with lower hip BMD, consistent with the role of secondary hyperparathyroidism in age-related bone loss. This finding underscores the importance of considering PTH—not only vitamin D—in the endocrine assessment of osteoporosis risk in older adults [24]. From a clinical standpoint, PTH may serve as a more stable indicator of chronic disturbances in calcium–vitamin D metabolism and skeletal turnover, particularly in populations with fluctuating vitamin D supplementation [25]. The coexistence of vitamin D deficiency, hypocalcaemia, and elevated PTH observed in this cohort reflects the well-established physiological response of the parathyroid axis to impaired calcium absorption. In older adults, reduced dietary intake, decreased intestinal calcium absorption, limited sunlight exposure, and impaired renal activation of vitamin D may all contribute to disturbances in mineral metabolism. Secondary hyperparathyroidism represents a compensatory response to maintain serum calcium levels but results in increased bone resorption and progressive skeletal fragility. Our findings demonstrate that these endocrine disturbances remain highly prevalent even in contemporary clinical care and may contribute substantially to bone loss in very old hospitalized patients.

The inverse association between serum 25-hydroxyvitamin D concentrations and hip BMD should be interpreted cautiously. Because of the cross-sectional design, circulating vitamin D levels most likely reflect current treatment status rather than cumulative vitamin D exposure during the development of osteoporosis. Patients with established osteoporosis or previous fractures are more likely to receive vitamin D supplementation and clinical monitoring, which may result in higher measured vitamin D concentrations despite persistently reduced bone mineral density (confounding by indication). In addition, residual confounding related to unmeasured clinical characteristics and the complex interrelationships among calcium metabolism parameters cannot be excluded. Consequently, this finding should not be interpreted as evidence that higher vitamin D concentrations adversely affect bone mineral density.

Interestingly, thyroid hormone replacement therapy was independently associated with higher hip BMD. This finding should not be interpreted as evidence of a protective effect of thyroid hormone replacement therapy. Residual confounding related to underlying thyroid disease, treatment status, follow-up, laboratory monitoring, multimorbidity, and healthcare utilization, which may translate into earlier detection and management of osteoporosis, cannot be excluded. This observation highlights the broader concept that structured chronic disease management—rather than isolated pharmacological effects—may influence skeletal outcomes in older adults [26, 27]. The association between higher serum TSH concentrations and lower hip BMD should be interpreted cautiously. Although thyroid dysfunction has been associated with skeletal health, particularly in the setting of suppressed TSH or overt hyperthyroidism, the mechanisms underlying the present finding remain uncertain. In this cohort, thyroid hormone replacement therapy most likely reflected treatment of hypothyroidism rather than hyperthyroidism. Residual confounding related to the underlying thyroid disease, treatment adequacy, frailty, multimorbidity, or non-thyroidal illness cannot be excluded. Therefore, this finding should be regarded as exploratory rather than evidence of a causal relationship.

Functional outcomes using routinely collected Comprehensive Geriatric Assessment, including TUG and Barthel Index scores, were primarily driven by age and living setting. Endocrine parameters were not independently associated with mobility in adjusted analyses, and endocrine–mobility associations could only be explored among community-dwelling participants due to missing data in nursing home residents [28]. More comprehensive assessments of frailty and sarcopenia, such as gait speed, handgrip strength, one-leg stance, or the Short Physical Performance Battery, were unavailable. These findings emphasize that while endocrine dysregulation contributes to skeletal health, functional mobility in very old adults is likely determined by a complex interplay of neuromuscular, cognitive, and environmental factors that extend beyond bone metabolism alone [29]. Future prospective studies should incorporate multidimensional functional assessments to further clarify the relationship between physical performance and skeletal health.

In addition, total protein levels were frequently at the lower end of the normal range, which may reflect nutritional vulnerability in this very old inpatient cohort. Malnutrition and reduced protein intake are well-recognized contributors to sarcopenia, frailty, and impaired bone health in older adults [30]. Although nutritional parameters were not the primary focus of the present study, these findings underline the multifactorial nature of skeletal vulnerability in advanced age, where endocrine disturbances interact with nutritional and functional factors [31, 32].

This study draws on a large, real-world cohort of very old adults, including both community-dwelling individuals and nursing home residents, reflecting routine care conditions in Germany. The simultaneous assessment of endocrine parameters, bone mineral density, mobility, and functional independence allows for an integrated evaluation of skeletal and functional ageing. Importantly, the focus on hip BMD as the primary outcome enhances clinical relevance, given its strong association with fracture risk and disability.

Several limitations should be acknowledged. First, the cross-sectional design precludes causal inference, particularly with respect to endocrine parameters and bone health. Second, substantial missing data—especially for lumbar spine BMD and TUG—limited the feasibility of multivariable analyses for certain outcomes and restricted endocrine–mobility analyses to community-dwelling participants. Third, information on supplementation and medication use was based on available records and may not fully capture dose, duration, or adherence. Furthermore, the complete-case approach may have introduced selection bias, as participants undergoing DXA assessment were likely healthier or sufficiently mobile to undergo imaging compared with those without available measurements. Consequently, the reported associations may not be fully generalizable to all very old hospitalized patients. Detailed information on previous or ongoing osteoporosis-specific pharmacological treatment, including bisphosphonates, denosumab, osteoanabolic agents, selective estrogen receptor modulators, or calcitonin, was not recorded in the available database. Consequently, these treatments could not be incorporated into the multivariable models, and residual confounding related to osteoporosis therapy cannot be excluded. Therefore, the observed associations between PTH, calcium metabolism, and hip BMD should be interpreted as observational and hypothesis-generating rather than as evidence of an independent causal effect.

Another important limitation is that only total serum calcium concentrations were available. Albumin concentrations and ionized calcium measurements were not consistently recorded and therefore albumin-adjusted calcium could not be calculated. Because hypoalbuminemia is common among hospitalized very old adults, some patients classified as hypocalcaemic based on total serum calcium may have had physiologically normal ionized calcium concentrations. Consequently, the operational prevalence of secondary hyperparathyroidism (13.7%) may have been modestly overestimated, and the observed associations should be interpreted with appropriate caution. Accordingly, calcium-related findings should be interpreted cautiously, whereas the association between elevated PTH and lower hip BMD appears more robust.

In addition, serum 25-hydroxyvitamin D concentrations were measured using an automated electrochemiluminescence immunoassay as part of routine clinical care rather than by liquid chromatography–tandem mass spectrometry (LC-MS/MS), which is considered the analytical reference method. Immunoassays may exhibit variable analytical accuracy, particularly at low vitamin D concentrations and in distinguishing between 25(OH)D₂ and 25(OH)D₃. Consequently, some degree of measurement variability cannot be excluded. Information regarding multivitamin use or biotin supplementation was unavailable. Because biochemical analyses were performed using automated electrochemiluminescence immunoassays, interference from high-dose biotin supplementation cannot be completely excluded, although the prevalence of clinically relevant biotin use in this hospitalized geriatric population is expected to be low. Although the cohort predominantly comprised patients admitted with acute fractures, fracture information was not available as a standardized outcome variable. In particular, the database did not reliably distinguish fragility from traumatic fractures or incident fractures from previous fracture history. Consequently, fracture-specific association analyses could not be performed. Finally, residual confounding by comorbidities and functional status cannot be excluded. Because this study intentionally included an unselected hospitalized geriatric population, patients with multiple comorbidities, including neurological, rheumatological, orthopedic, and other mobility-limiting conditions, were not excluded. Although this approach improves the real-world applicability of the findings, these conditions may have influenced bone mineral density, vitamin D status, and functional performance, thereby contributing to residual confounding.

Our findings carry important implications for the care of older adults. Osteoporosis risk and functional decline should be addressed proactively in both community and institutional settings. Reliance on vitamin D levels alone may be insufficient for risk stratification, whereas PTH may provide additional clinically relevant information [33].

## Conclusion

In this very old population, skeletal and functional health were driven primarily by biological ageing and endocrine dysregulation rather than by living setting alone. Despite their higher age, nursing home residents did not exhibit substantially worse bone mineral density or endocrine profiles compared with community-dwelling older adults, underscoring the need for individualized osteoporosis risk assessment across care settings. Parathyroid hormone emerged as a robust marker of lower hip bone mineral density, whereas isolated vitamin D measurements should be interpreted cautiously in routine care. Functional mobility and independence were largely determined by age and care context, with limited contribution from endocrine parameters. These findings emphasize that effective osteoporosis prevention and functional preservation in older adults require comprehensive, patient-centred care strategies that extend beyond residential status and single laboratory values.

### Vitamin D, calcium, PTH, and secondary hyperparathyroidism

Vitamin D deficiency was highly prevalent in this very old inpatient cohort. Among participants with available vitamin D measurements, 605 (68.6%) had vitamin D levels below 20 ng/mL (95% CI: 65.2–70.8), and 401 (45.5%) had levels below 10 ng/mL (95% CI: 44.1–50.9).

Disturbances in calcium homeostasis were also common. Hypocalcaemia was present in 39.1% of participants (442/1,130; 95% CI: 36.2–42.1), while elevated parathyroid hormone (PTH) levels (> 65 pg/mL) were observed in 29.9% (296/990; 95% CI: 27.1–32.9). The combination of hypocalcaemia and elevated PTH—used as an operational definition of secondary hyperparathyroidism—was identified in 13.7% of participants (135/988; 95% CI: 11.6–16.0) (Supplementary Fig S1).

Consistent with the physiological relationship between vitamin D and parathyroid hormone, PTH concentrations decreased across increasing vitamin D categories. Patients with severe vitamin D deficiency (< 10 ng/mL) exhibited the highest PTH levels (median 52.0 pg/mL, IQR 39.8–71.3), whereas patients with vitamin D levels ≥ 30 ng/mL had substantially lower PTH concentrations (median 36.4 pg/mL, IQR 27.0–53.2).

These endocrine abnormalities were associated with skeletal outcomes. Participants with elevated PTH levels had significantly lower hip bone mineral density compared with those without PTH elevation (median T-score − 2.3 vs. − 1.9, *p* = 0.004). In adjusted analyses including age and sex, elevated PTH remained independently associated with lower hip BMD (β − 0.23, 95% CI − 0.45 to − 0.01; *p* = 0.042) (Table 1).

**Table 1:** Relationship between vitamin D status, parathyroid hormone (PTH), and calcium concentrations. **(A)** Distribution of parathyroid hormone (PTH) levels across categories of serum 25-hydroxyvitamin D. Lower vitamin D levels were associated with higher PTH concentrations, consistent with secondary hyperparathyroidism due to vitamin D deficiency. **(B)** Serum calcium concentrations according to vitamin D categories. Calcium values are presented as mean ± standard deviation and median (interquartile range). Vitamin D categories were defined as < 10 ng/mL, 10–20 ng/mL, 20–30 ng/mL, and ≥ 30 ng/mL

**A**			
**Vitamin D (ng/mL)**	**n**	**Mean PTH ± SD**	**Median PTH (IQR)**
< 10	370	59.9 ± 31.7	52.0 (39.8–71.3)
10–20	182	55.0 ± 53.4	45.1 (35.6–60.5)
20–30	94	49.9 ± 31.6	42.1 (31.0–57.8)
≥ 30	109	44.9 ± 29.7	36.4 (27.0–53.2)
**B**			
**Vitamin D (ng/mL)**	**n**	**Mean Ca ± SD**	**Median Ca (IQR)**
< 10	399	2.19 ± 0.14	2.19 (2.10–2.26)
10–20	203	2.21 ± 0.13	2.21 (2.12–2.29)
20–30	104	2.21 ± 0.13	2.18 (2.13–2.30)
≥ 30	115	2.24 ± 0.13	2.23 (2.16–2.33)

In contrast, vitamin D levels alone did not show a consistent positive association with hip BMD in multivariable models. Participants receiving vitamin D supplementation had higher circulating vitamin D levels and lower PTH concentrations but did not exhibit higher hip BMD compared with non-supplemented individuals, suggesting potential confounding by indication, as supplementation is more frequently prescribed to patients with established osteoporosis or secondary hyperparathyroidism.

Serum total protein concentrations were within the lower normal range in many participants, reflecting the high prevalence of frailty and potential nutritional vulnerability in this very old inpatient population. Mean total protein levels were 60.97 ± 7.39 g/L, Median (IQR): 61 (56–66), with a proportion of patients presenting values below the reference range.

### Multivariable associations with hip bone mineral density

To assess independent determinants of bone health in this very old population, multivariable linear regression analyses were performed with hip BMD as the primary skeletal outcome **(**Table 2**)**.

**Table 2:** Multivariable determinants of hip bone mineral density

**Predictor**	β (95% CI)	*p*-value
Age	−0.046 (− 0.063 to − 0.029)	**< 0.001**
Female sex	−0.150 (− 0.420 to 0.120)	0.28
Nursing home residence	−0.326 (− 0.858 to 0.207)	0.23
Vitamin D (ng/ml)	−0.013 (− 0.023 to − 0.002)	**0.016**
Calcium (mmol/l)	0.650 (− 0.212 to 1.51)	0.14
PTH (pg/ml)	−0.006 (− 0.010 to − 0.002)	**0.002**
Vitamin D supplementation	−0.113 (− 0.493 to 0.266)	0.56
Calcium supplementation	0.101 (− 0.556 to 0.758)	0.76
Thyroid hormone replacement therapy	0.380 (0.098 to 0.661)	**0.008**

**Model fit**: Adjusted R² = 0.133, F = 7.37, *p* = 6.7 × 10⁻¹⁰, No relevant multicollinearity (VIF < 1.2)

The overall model was statistically significant (F = 7.37, p = < 0.001) and explained 15.4% of the variance in hip BMD (adjusted R² 0.133). No evidence of problematic multicollinearity was observed.

Increasing age was strongly associated with lower hip BMD (β − 0.046 per year, 95% CI: −0.063 to − 0.029; *p* < 0.001). Higher PTH levels were independently associated with lower hip BMD (β − 0.006, 95% CI: −0.010 to − 0.002; *p* = 0.002). In contrast, serum Vitamin D levels were also significantly associated with hip BMD (β − 0.013, 95% CI: −0.023 to − 0.002; *p* = 0.016), showing a small inverse association with hip BMD, but vitamin D supplementation was not independently associated with hip BMD (β = −0.113, 95% CI − 0.493 to 0.266; *p* = 0.56). Likewise, calcium supplementation showed no independent association with hip BMD (β = 0.101, 95% CI − 0.556 to 0.758; *p* = 0.76). Participants receiving vitamin D supplementation had higher circulating vitamin D levels and lower PTH concentrations but did not show higher hip BMD compared with non-supplemented participants.

After adjustment for age and endocrine parameters, living setting was not independently associated with hip BMD (nursing home vs. community: β − 0.326, 95% CI: −0.858 − 0.207; *p* = 0.23), nor was sex (female vs. male: β − 0.150, 95% CI: −0.420 − 0.120; *p* = 0.28). Interestingly, thyroid hormone replacement therapy was independently associated with higher hip BMD (β 0.38, 95% CI: 0.098 to 0.661; *p* = 0.008).

Overall, these findings indicate that age and disturbances in calcium–PTH homeostasis represent the strongest endocrine determinants of hip bone mineral density in this very old population, whereas living setting and supplementation status showed no independent associations.

Consistent with the predefined analytical strategy, hip BMD was used as the main densitometric outcome because it provides a robust and clinically relevant measure of skeletal status in very old adults and is less affected by degenerative spinal changes than lumbar spine measurements.

### Mobility and functional outcomes

#### Timed Up and Go (TUG)

Among participants with available TUG measurements, community-dwelling individuals had a mean TUG of 24.0 ± 21.4 s (median 18, IQR 13–26), while nursing home residents had a mean TUG of 20.2 ± 10.6 s (median 18, IQR 13–26.2).

In multivariable analyses adjusting for age and sex, living setting was not independently associated with TUG performance (nursing home vs. community: β − 2.90, 95% CI: 18.4 − 24.2; *p* = 0.79). Neither age nor sex was significantly associated with TUG in this model.

### Sensitivity analysis excluding supplement users

In sensitivity analyses excluding participants receiving vitamin D and/or calcium supplementation, associations between endocrine parameters and mobility remained directionally similar. Vitamin D showed a non-significant trend toward a positive association with log-transformed TUG (β: 0.021, 95% CI: 0.003 − 0.046; *p* = 0.088), while calcium and PTH remained non-significant. Overall model fit was modest (adjusted R² 0.015).

### Barthel Index

Functional independence as assessed by the Barthel Index was available for the majority of participants. In adjusted analyses, Barthel scores were primarily driven by age and living setting, while endocrine parameters did not show independent associations with functional independence after adjustment.

### Exploratory thyroid analyses

Exploratory analyses were performed to evaluate potential associations between thyroid function parameters and hip bone mineral density (BMD). In models including thyroid hormone levels, neither free thyroxine (fT4) nor free triiodothyronine (fT3) showed independent associations with hip BMD after adjustment for age, sex, and thyroid medication use (Supplementary Figs. S2, S3).

When thyroid-stimulating hormone (TSH) levels were analyzed categorically, a non-linear pattern was observed. Both suppressed TSH levels (< 0.4 mIU/L) and elevated TSH levels (> 4 mIU/L) were associated with lower hip BMD compared with the euthyroid range (0.4–4 mIU/L). Mean hip BMD values were − 2.15 ± 1.12 for suppressed TSH, − 1.92 ± 1.18 for euthyroid TSH, and − 2.15 ± 0.97 for elevated TSH, suggesting a U-shaped relationship between TSH and skeletal health (Supplementary Fig. S4).

Among participants receiving thyroid medication with available TSH measurements (*n* = 256), 33 individuals (12.9%) had suppressed TSH levels (< 0.4 mIU/L). Median TSH values were slightly higher in patients receiving thyroid medication compared with those without thyroid medication (median 1.69 mIU/L [IQR 0.79–3.00] vs. 1.30 mIU/L [IQR 0.78–2.09], respectively) (Table 3).

**Table 3:** Thyroid function parameters and hip bone mineral density.** (A)** Median thyroid-stimulating hormone (TSH) levels among patients receiving thyroid medication compared with those without thyroid medication. **(B)** Hip bone mineral density (BMD, T-score) according to thyroid medication status. **(C)** Hip bone mineral density across TSH categories (< 0.4, 0.4–4, and > 4 mIU/L). Both suppressed and elevated TSH values were associated with lower BMD compared with the euthyroid range, suggesting a non-linear (U-shaped) relationship between thyroid function and bone health

**A**			
**Group**	*n*	**Median TSH (IQR)**	
Thyroid medication	324	**1.69 (0.79–3.00)**	
No thyroid medication	878	**1.30 (0.78–2.09)**	
**B**			
**Group**	**N**	**Median Hip BMD (IQR)**	
Thyroid medication	138	**−1.85 (− 2.50 to − 1.13)**	
No thyroid medication	420	**−2.10 (− 2.80 to − 1.30)**	
**C**			
**TSH category**	**n**	**Mean BMD ± SD**	**Median BMD**
< 0.4	58	−2.15 ± 1.12	−2.1
0.4–4	446	−1.92 ± 1.18	−2.0
> 4	39	−2.15 ± 0.97	−2.1

Overall, thyroid hormone parameters did not show consistent independent associations with hip BMD after adjustment for age and medication use. These findings suggest that thyroid-related markers may play a secondary role compared with disturbances in the vitamin D–calcium–PTH axis in determining skeletal health in this very old population.

## Supplementary Information

Below is the link to the electronic supplementary material.

Supplementary Material 1

## Data Availability

The datasets generated and/or analysed during the current study are available from the corresponding author on reasonable request, subject to institutional approval and applicable data protection regulations.
