# Supplementary material for: Secondary hyperparathyroidism and lower hip bone mineral density in very old hospitalized adults: a real-world endocrine bone study
Source: BMC Endocr Disord. 2026 Aug 6;26:225. doi: 10.1186/s12902-026-02456-y (PMC13445846; doi:10.1186/s12902-026-02456-y)
Supplement: Supplementary file 1 [file 12902_2026_2456_MOESM1_ESM.docx]

**Supplementary Material**

**Supplementary Figure S1.** Relationship between serum vitamin D levels and hip bone mineral density, color-coded by parathyroid hormone (PTH) levels. Higher PTH concentrations were associated with lower hip BMD across the range of vitamin D values, whereas vitamin D levels alone showed no consistent positive association with bone density.

**A**

**B**

**Supplementary Figures S2 (A, B).** **Regression of Hip and Lumbar Spine BMD on TSH Levels**. A) This scatter plot with a fitted regression line shows the relationship between TSH levels and hip BMD. B) This scatter plot with a fitted regression line illustrates the relationship between thyroid-stimulating hormone (TSH) levels and lumbar BMD.

**Supplementary Figure S3. Correlation Matrix of Biochemical Markers and BMD**. This heatmap displays the correlation coefficients between key biochemical markers (Vitamin D, TSH, fT3, fT4, PTH, and Calcium).

**Supplementary Figure S4.** Hip bone mineral density across thyroid-stimulating hormone (TSH) categories ordered from low to normal to high.
